# Zona Pellucida Domain-Containing Protein β-Tectorin is Crucial for Zebrafish Proper Inner Ear Development

**DOI:** 10.1371/journal.pone.0023078

**Published:** 2011-08-02

**Authors:** Chung-Hsiang Yang, Chia-Hsiung Cheng, Gen-Der Chen, Wei-Hao Liao, Yi-Chung Chen, Kai-Yun Huang, Pung-Pung Hwang, Sheng-Ping L. Hwang, Chang-Jen Huang

**Affiliations:** 1 Institute of Biochemical Sciences, National Taiwan University, Taipei, Taiwan; 2 Institute of Biological Chemistry, Academia Sinica, Taipei, Taiwan; 3 Institute of Cellular and Organismic Biology, Academia Sinica, Taipei, Taiwan; Texas A&M University, United States of America

## Abstract

**Background:**

The zona pellucida (ZP) domain is part of many extracellular proteins with diverse functions from structural components to receptors. The mammalian β-tectorin is a protein of 336 amino acid residues containing a single ZP domain and a putative signal peptide at the N-terminus of the protein. It is 1 component of a gel-like structure called the tectorial membrane which is involved in transforming sound waves into neuronal signals and is important for normal auditory function. β-Tectorin is specifically expressed in the mammalian and avian inner ear.

**Methodology/Principal Findings:**

We identified and cloned the gene encoding zebrafish β-tectorin. Through whole-mount *in situ* hybridization, we demonstrated that *β-tectorin* messenger RNA was expressed in the otic placode and specialized sensory patch of the inner ear during zebrafish embryonic stages. Morpholino knockdown of zebrafish β-tectorin affected the position and number of otoliths in the ears of morphants. Finally, swimming behaviors of β-tectorin morphants were abnormal since the development of the inner ear was compromised.

**Conclusions/Significance:**

Our results reveal that zebrafish *β-tectorin* is specifically expressed in the zebrafish inner ear, and is important for regulating the development of the zebrafish inner ear. Lack of zebrafish *β-tectorin* caused severe defects in inner ear formation of otoliths and function.

## Introduction

The zona pellucida (ZP) was first discovered as a glycoprotein surrounding the plasma membrane of an oocyte, and it is important in fertilization. The ZP domain is a sequence shared by many extracellular proteins with diverse functions from structural components to receptors. Among these proteins are the mammalian ZP1, ZP2, and ZP3, and non-mammalian egg-coating proteins, Tamm-Horsfall protein (THP), glycoprotein (GP)-2, α- and β-tectorins, transforming growth factor (TGF)-β receptor III, endoglin, deleted in malignant brain tumor (DMBT)-1, no-mechanoreceptor potential-A (Nomp A), Dumpy, and cuticlin-1 [1]. Each of these ZP-containing proteins is composed of a signal sequence driving these proteins to the endoplasmic reticulum (ER), and each possesses a ZP domain of approximately 260 amino acids long that is comprised of 8∼10 conserved cysteine residues, a C-terminal, a hydrophobic transmembrane-like region, and a short cytoplasmic tail [2], [3]. ZP domain-containing proteins are highly conserved among all species and are often glycosylated [4]. They are generally modified with a variable number of high-mannose type, N-linked oligosaccharides in the ER. These proteins can be further modified by the addition of O-linked oligosaccharides and by processing of high-mannose-type, N-linked oligosaccharides to the complex type when transferred to Golgi apparatuses. ZP domain-containing proteins are often present in filaments and/or matrices which play important roles in protein polymerization [1].

In the inner ear organ of Corti, the tip of hair cell stereocilium bundles is covered by a gel-like matrix called the tectorial membrane. The mammalian tectorial membrane is formed by 3 different collagens (types II, V, and IX) combined with 3 non-collagenous, glycosylated polypeptides, called *α*-tectorin, *β*-tectorin, and otogelin [1]. The tectorial membrane is a particular structure that deflects the stereocilia of hair cells during sound-triggered vibrations of the basilar membrane, and hair cells facilitate the transduction of sounds into neural signals. *α*- and *β*-tectorins belong to the ZP domain-containing protein family, and mutations in *α*-tectorin or β-tectorin were reported to result in human nonsyndromic deafness. Studies of the human *α*-tectorin gene, *TECTA*, showed that it is related to dominant forms of prelingual, nonprogressive deafness: DFNA8 (MIM601543) and DFNA12 (MIM601842) [5] or a recessive form at locus DFNB21 [6]. On the other hand, *TECTB*, which encodes β-tectorin, also plays an important role in maintaining the normal function of the tectorial membrane. Previous studies on knockout mice reported that the structure of the striated-sheet matrix is disrupted and cochlear tuning is sharpened in *TECTB* ^−/−^ mice [7]. Together, both types of tectorins are important for maintaining normal auditory function of the inner ear.

Currently, only mammalian and chicken tectorins have been identified and cloned [8]. Little is known about zebrafish β-tectorin and its role during embryonic development. Zebrafish have many benefits as a model animal for studying genes involved in inner ear development: first, zebrafish eggs develop *in vitro*; second, the zebrafish ear is transparent for the first few weeks of life; and third, forward genetic screens and antisense technology are well-established [9]. The zebrafish inner ear system consists of semicircular canals, otoliths, and different sensory patches that are formed by special type of cells, called hair cells. The semicircular canals are attached to the sensory epithelium called cristae which are located at the base of each canal. Cristae are important for sensing the position of the head and angular acceleration. Polycrystalline masses called otoliths are connected to 2 or more macular organs, called saccules and utricles [9]. Zebrafish is considered to be a fish with excellent hearing in the teleost family due to the function of Weberian ossicles which connect the swim bladder to the saccule allowing sound amplification [10], [11]. Although, the structure of the zebrafish inner ear greatly differs from that of the mammalian inner ear due to the lack of a cochlea, the convenience in handling it and the evolutionarily conserved molecular mechanisms of inner ear development make zebrafish a good animal model for studying development of the inner ear.

In order to investigate the role of ZP domain-containing proteins in zebrafish inner ear development, we predicted and identified a zebrafish ZP domain-containing protein, zebrafish β-tectorin, through a bioinformatics method, and designed a specific morpholino to knock down the expression of zebrafish β-tectorin. We also characterized the temporal and spatial expressions of zebrafish β-tectorin by whole-mount *in situ* hybridization, and demonstrated that it is specifically expressed in the zebrafish inner ear in the early stages of development. Morpholino knockdown of β-tectorin expression in zebrafish resulted in abnormal inner ear development. Otoliths of β-tectorin morphants showed delocalization or a fused pattern. Taken together, we propose that the zebrafish β-tectorin plays an important role in the proper formation of zebrafish inner ear components, and therefore a lack of it will cause serious defects in development of the inner ear.

## Results

### Cloning of *β-tectorin* from zebrafish

**Figure 1:**
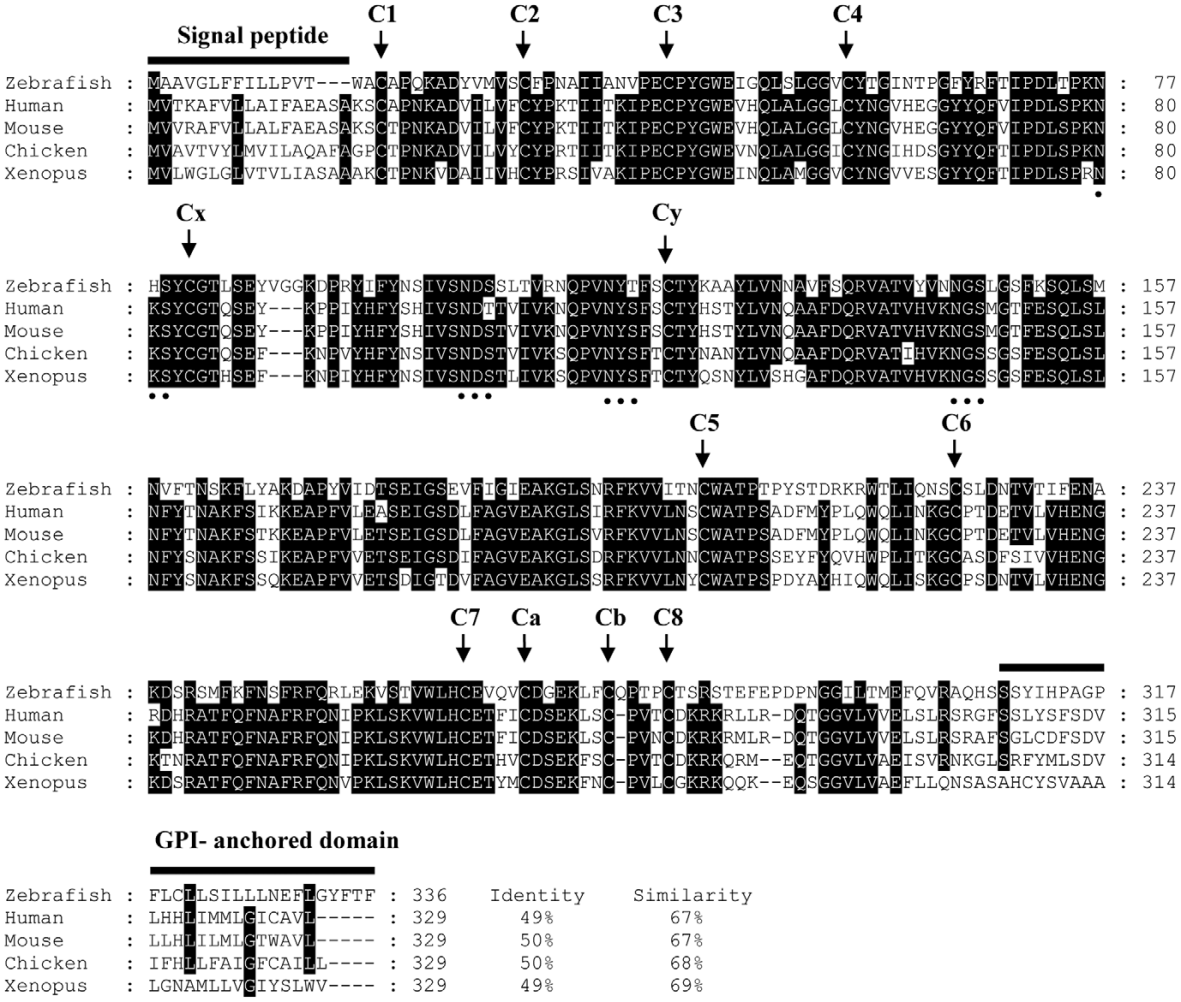
Zebrafish β-tectorin amino acid sequence alignment with those of other species. Deduced amino acid sequences of zebrafish β-tectorin were aligned with those of human, mouse, chicken, and *Xenopus*. All β-tectorin proteins contained a conserved zona pellucida (ZP) domain of approximately 260 amino acids, with 12 highly conserved cysteine residues (indicated by arrows). Identical residues in 4 or 5 proteins are highlighted. Signal peptide and putative GPI-anchored domains are heavily overlined. The putative N-linked glycosylation sites are indicated by dots (**…**). The accession numbers of each β-tectorin from different species are listed below: human (XM_521604), mouse (X99806), chicken (AAA92461), and *Xenopus* (CAJ82963).

The overall deduced amino acid sequences of zebrafish β-tectorin respectively showed 49%, 50%, 50%, and 49% identities to those of human, mouse, chicken, and *Xenopus* β-tectorin (Fig. 1). The zebrafish β-tectorin protein contains a conserved ZP domain, which has highly conserved cysteines of C1 to C8, and Cx, Cy, Ca, and Cb. A signal peptide of 16-amino acids, MAAVGLFFILLPVTWA, in the NH_2_-terminal was predicted by the online software, SignalP 3.0 (http://www.cbs.dtu.dk/services/SignalP/), which likewise occurs in mammalian β-tectorin proteins. Mammalian β-tectorin proteins have a signal peptide of 17 amino acids. A hydrophobic C-terminus characteristic of proteins that are membrane bound via a putative GPI-anchor was reported in avian and mammalian β-tectorins [12], [13]. Moreover, human β-tectorin is a glycoprotein and contains 4 N-linked sugar chains on Asn80, Asn104, Asn116, and Asn145. Similarly, the zebrafish β-tectorin protein contains 4 N-linked glycosylation sites of ^77^NHS, ^104^NDS, ^116^NYT, and ^145^NGS (Fig. 1).

### Genomic structure of the zebrafish β-tectorin gene

**Figure 2:**
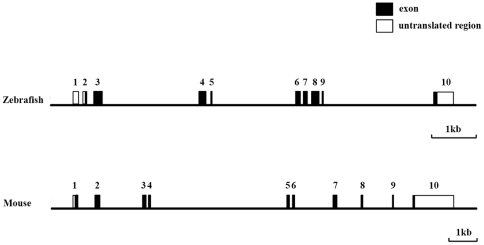
Genomic organization of zebrafish and mouse *β-tectorin* genes. Coding regions are shown as filled boxes numbered from 1 to 10 in both zebrafish and mouse *β-tectorin* genes. The 5′- and 3′-untranslated regions are shown as open boxes, while introns and 5′- and 3′-flanking regions are indicated by solid lines.

We then used the 1542 bp of zebrafish *β-tectorin* cDNA (with accession no. FJ374270) to perform an online BLAST search of the GenBank database. The zebrafish β-tectorin cDNA matched 10 non-contiguous regions of a 92,355-bp zebrafish BAC clone, CH73-92E20 (GenBank accession no. CU462848). Subsequently, a BLAST 2-sequence comparison of BAC CH73-92E20 with the zebrafish β-tectorin cDNA indicated that *β-tectorin* cDNA contained 10 putative exons and 9 introns spanning at least 8.6 kb (Fig. 2). Using these putative exons as a model, a sequence alignment was produced such that each intron concurred with the GT/AG intron donor/acceptor site rule [14]. Exon 1 contained the 5′-UTR, while exon 2 contained the putative translation initiation site. Exon 2 contained 9 bp of the 5′-UTR and 66 bp of the first coding sequences of *β-tectorin* cDNA. Exon 10 contained the last 69 bp of the coding sequences and 362 bp of 3′-UTR. The size of the introns considerably varied, ranging 81 (intron 8) to 2419 bp (intron 9).

Comparison of the exon-intron organization of zebrafish and mouse *β-tectorin* genes indicated that their genomic structures were similar with 10 exons and 9 introns. The mouse *β-tectorin* gene spanned approximately 15.4 kb. In addition, the average intron size of the mouse *β-tectorin* gene (1411 bp) was larger than that of the zebrafish *β-tectorin* gene (799 bp) (Fig. 2).

### Expression profiles of zebrafish *β*-*tectorin* messenger (m)RNA in adult tissues and embryos at different developmental stages

**Figure 3:**
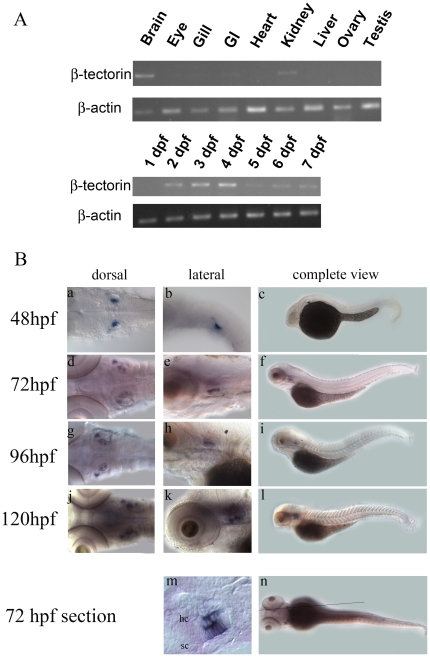
Expression profiles of zebrafish *β-tectorin* mRNAs by RT-PCR and whole-mount *in situ* hybridization. (A) RT-PCR of the *β-tectorin* transcript was performed using a pair of primers to produce a DNA fragment of 1208 bp. β-Actin bands were also used to normalize the amount of cDNA prepared from different tissues and embryos at different developmental stages. (B) Whole-mount *in situ* hybridization with antisense *β-tectorin* at different developmental stages was performed. The images were taken from the dorsal (a, d, g, j) and the lateral view (b, e, h, k), and complete lateral view (c, f, i, l) with the anterior to the left and dorsal to the top. Longitudinal sections of the embryo were at 72 hpf with anterior to the left and dorsal to the top (panel m). The straight line in panel n represents the region of sections in panel m. hc, hair cell; sc, supporting cell.

Expression levels of zebrafish *β*-*tectorin* transcripts in adult tissues and embryos from different developmental stages were examined by an RT-PCR analysis. A pair of primers was used to amplify a DNA fragment that spanned exons 2 to 4 to avoid genomic DNA interference in the PCRs. The amplified product of this pair of primers was about 1208 bp long. As shown in Fig. 3A, a high level of *β-tectorin* expression was detected in the brain, with moderate expression in the kidneys and less in the intestines.

During embryogenesis, *β*-*tectorin* transcripts were not detected at 1 day post-fertilization (dpf), and their expression levels constantly increased thereafter; however, a significant decrease in the expression level was observed at 5 dpf and thereafter (Fig. 3A).

Spatial and temporal expression patterns of zebrafish *β*-*tectorin* were further analyzed by whole-mount *in situ* hybridization. During different stages of development, expression of the *β*-*tectorin* transcript was specifically detected in the anterior and posterior maculae on both sides of the zebrafish from 48∼120 h post-fertilization (hpf) (Fig. 3B, panels a∼l). It was interesting to note that the expression of *β*-*tectorin* mRNA in the anterior macula was much weaker than that of the posterior macula in 48-hpf embryos. The signals of *β*-*tectorin* in situ hybridization are restricted to the macula of the inner ear, no signals in other parts of the embryos can be detected in various stages (Fig. 3B, panels c, f, i and l). Through longitudal section of the zebrafish inner ear at 72 hpf, *β*-*tectorin* is both expressed in the hair cells and supporting cells of macula, and the expression pattern resembles the early expression of *pax5* in the macula [15]. The overall expression pattern of zebrafish *β*-*tectorin* in the inner ear was quite similar to that of the *Starmaker* gene, which is also expressed in the anterior and posterior maculae on both sides of the inner ear [16].

### Abnormal otolith formation in *β*-*tectorin* morphants

Morpholino (MO)-mediated knockdown of genes in zebrafish embryos has become a routine and efficient method to provide information about gene function *in vivo* [17]. To examine the function of *β-tectorin in vivo*, we designed zebrafish *β*-*tectorin* MOs, which targeted the sequence located at the 5′-UTR of *β*-*tectorin* mRNA, to specifically knock down the translation of endogenous *β*-*tectorin* mRNA.

To determine the specificity of the MO used, a control approach was used. The 25-bp target sequence of the *β*-*tectorin* MO was cloned upstream of the green fluorescent protein (GFP) ORF into a pCMV backbone expression vector (bTec-GFP). As for the control, a target sequence containing 5 mismatches was used (MM b-Tec-GFP). The MM b-Tec-GFP or bTec-GFP plasmid was injected into zebrafish embryos in the absence or presence of a 9-ng *β*-*tectorin* MO. Co-injection of bTec-GFP RNA and the *β*-*tectorin* MO completely blocked GFP expression (*n* = 22/24; Fig. S1A). Conversely, GFP expression was not affected when 5 bp of the target sequence was exchanged (MM b-Tec-GFP) (*n* = 36/36; Fig. S1B) indicating the specificity of the *β*-*tectorin* MO.

**Figure 4:**
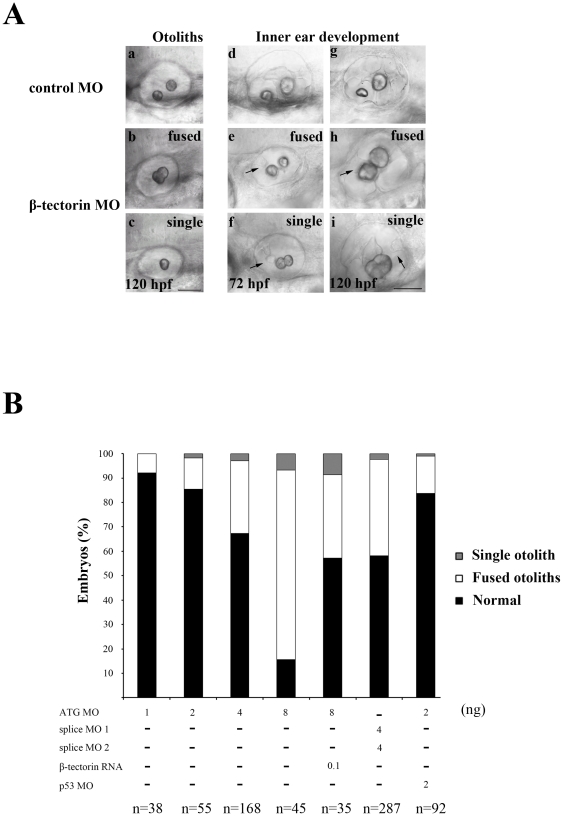
Abnormal otolith phenotypes in *β-tectorin* morphants. (**A**) The otolith phenotypes of *β-tectorin* morphants are classified into normal (normal, panel a), fused (fused, panel b) and single otoliths (single, panel c). Abnormal development of the vestibular system is shown by arrows in *β-tectorin* morphants from 72 to 120 hpf (panels d to i). (B) The percentage of abnormal otolith phenotypes in zebrafish embryos injected with different *β-tectorin* MOs or combined with *β-tectorin* mRNAs or p53 MOs. All samples are observed at 72 hpf. Bars, 50 µm.

After injecting 4 ng/embryo of *β*-*tectorin* MO, we observed abnormal otolith morphology in embryos at 120 hpf. There are 2 otoliths on each side in wild-type (WT) zebrafish embryos; the one in the anterior has a flattened-oval shape, whereas the other one in the posterior, is larger, and has a round shape (Fig. 4A, panel a). *β*-*Tectorin* morphants displayed 2 different phenotypes including the fusion of 2 otoliths (Fig. 4A, panel b, *n* = 50/168, 29%; Fig. 4B) as well as a single otolith (Fig. 4A, panel c, *n* = 5/168, 2.9%; Fig. 4B). Changes in morphology of the otoliths were correlated with the irregular formation of the vestibular system in the inner ear; development of semicircular canals seemed to be affected in *β*-*tectorin* morphants when observed at 72∼120 hpf. *β*-*Tectorin* morphants with either fused or single otoliths were observed with abnormal semicircular canal outgrowth (Fig. 4A, panels e, h, f, and i, arrows), whereas the control showed 2 normal otoliths and outgrowth of the semicircular canals (Fig. 4A, panels d and g).

We also generated splice MOs to block splicings of the *β*-*tectorin* sequence within exon 2 and exon 3 (supplementatry Fig. 2A). The splice MO 1 which blocks a splicing donor site locates at the boundry of Exon 2 and intron 2 while the splice MO 2 targets the acceptor site at the boundry of intron 2 and Exon 3. Each of the splice MOs was used individually, producing abnormal phenotypes similar to those of the ATG MOs, differing only in efficiency (data not shown). The combination of the two splice MOs is more efficient in generating abnormal phenotypes that include both the fused and single otolith phenotype like the ATG MO. The efficiency of the splice MO mixture was analyzed by RT-PCR (supplementary Fig. 2B). After injecting a mixture of splice MOs 2 ng each, we observed the appearance of the fusion of 2 otoliths (n = 113/287, 39.37%; Fig. 4B) and single otolith (n = 7/287, 2.43%; Fig. 4B) in the resulting morphants. There were no other severe morphological defects in the ATG MO (supplementary Fig. 3). Gradient increases in the amount of ATG MO injected into the embryos also showed increases in the abnormal phenotypes in a dosage-dependent manner (Fig. 4B).

To rule out the possibilities that the phenotypes of these morphants are the results of off-target effect of the morpholino used [18], the p53 MO was used in co-injecting with ATG MO into embryos and the phenotypes of these embryos were observed. The percentage of abnormalities in the *β*-*Tectorin* morphants coinjected with p53 MO was approximately equal to that of *β*-*Tectorin* morphants (Fig. 4B), suggesting that the phenotypes of these morphants were not related to off-target effect of the morpholino.

To further confirm the specificity of gene knockdowns by the *β-tectorin* MO, mRNA rescue was performed. Full length *β*-*Tectorin* mRNAs, which were synthesized in vitro and injected into embryos in one- or two- cell stage, were used to investigate the rescue of *β*-*Tectorin* morphants. Misexpressions of the *β*-*Tectorin* mRNA have no effect on morphologies of the control embryos without MO injection. Coinjection of 8 ng ATG MO with ∼100 pg *β*-*Tectorin* mRNA into each embryo resulted in a reduction in the percentage of the *β*-*Tectorin* morphant with abnormal ear phenotypes, fused otoliths (n = 12/35, 34%) and single otolith (n = 3/35, 8.5%), whereas injection of 8 ng ATG MO alone showed a percentage of as high as 84% abnormalities in the inner ear, fused otoliths (n = 35/45, 78%), single otolith (n = 3/45, 6%) (Fig. 4B). These results demonstrated that the *β*-*Tectorin* mRNA can rescue defects in *β*-*Tectorin* morphants, validating the specificity of *β*-*Tectorin* MO.

### Development of the inner ear was affected in the *β*-*tectorin* MO morphants as shown by whole-mount *in situ* hybridization

**Figure 5:**
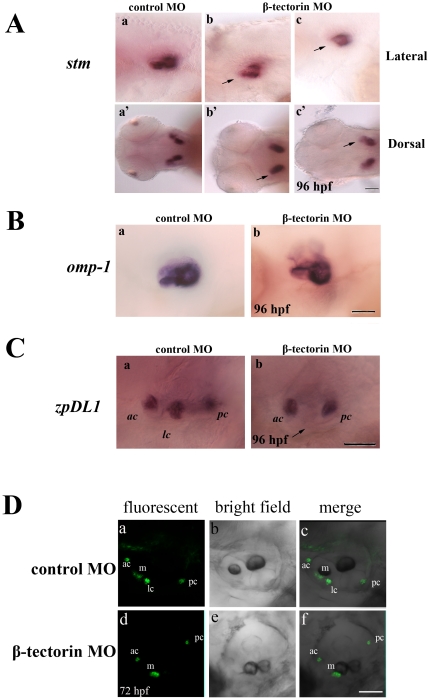
Characterization of ear defects in *β-tectorin* morphants. The expression levels of the following inner ear marker genes, such as starmaker (*stm*) (A), otolith matrix protein 1 (*omp-1*) (B), and zona pellucida-like domain-containing protein-like 1 (*zpDL1*) (C), were examined by whole-mount *in situ* hybridization in *β-tectorin* morphants. *Stm* signals in the anterior macula (am) of *β-tectorin* morphants decreased or even disappeared in fishes with either fused (panels b and b′) or single otoliths (panels c and c′), as indicated by arrows. *zpDL1* signals in the lateral crista (lc) of *β-tectorin* morphants vanished. Black bars, 100 µm (D) Confocal microscopy image analysis of *β*-tectorin morphants injected with FM1-43 dyes into the otic vesicle at 72 hpf. After injection, hair cells in anterior crista (ac), lateral crista (lc), macula (m) and posterior crista (pc) of control MO-injected embryos can take up FM1-43 dyes. White bar, 50 µm.

To further analyze inner ear defects observed in morphants, whole-mount *in situ* hybridization was first performed with *Starmaker* (*stm*), which was reported to regulate the growth, shape, and crystal lattice of otoliths [15]. In control MO-injected zebrafish, the *stm* transcript was expressed in anterior and posterior maculae on both sides at 96 hpf (Fig. 5B, lateral, panel a, dorsal panel a′). However, the *stm* transcript was less expressed in the anterior macula of *β*-*tectorin* morphants either with fused otoliths or a single otolith compared to control MO-injected zebrafish (Fig. 5B, panels b, b′ c, and c′; arrows). Otolith matrix protein 1 (*omp-1*) is important for otolith growth and correct anchoring of otoliths to the maculae [19]. The expression pattern of *omp-1* in *β*-*tectorin* morphants was also reduced in the anterior macula compared to the control, and its distribution seemed to differ from that of control MO-injected zebrafish (Fig. 5C). On the other hand, another gene marker, *zona pellucida-like domain-containing protein-like 1 (zpDL1)* (Genbank accession no. XM_00192195), was used to label anterior, lateral, and posterior cristae of otoliths of control MO-injected zebrafish at 96 hpf (Fig. 5D, panels a and b). In *β*-*tectorin* morphants with fused otoliths, the *zpDL1* signal was detected only in the anterior and posterior cristae of the inner ear, and the signal in the lateral crista was lost (Fig. 5D, arrow).

FM1-43, a fluorescent dye, is known for labeling hair cells of the inner ear by entering the mechanotransduction channels [20]. This function of FM1-43 allows us to monitor the formation of active hair cells in the morphants. For this purpose, zebrafish at diffenerent stages were injected with FM1-43 dyes specifically in lumen of the otic vesicle through an injection tube. FM1-43 dyes, which were taken up by macula and cristae, could be easily observed under a confocal fluorescence microscopy [20]. ATG MO-injected zebrafish embryos were further injected with FM1-43 dyes at 72 hpf to investigate whether the functions of these hair cells are affected. β-tectorin morphants with fused otoliths of 72 hpf had lost its lateral crista as compared to control zebrafish. These results are consistent with the data gained from whole-mount in situ hybridization, as signals of *zpDL1* lost in the lateral crista in the β-tectorin morphants.

Taken together, the altered expression patterns observed in ear-marker genes indicated that β-tectorin may play an important role in both otolith and inner ear formation during zebrafish development.

### Behavioral defects in β-tectorin morphants

**Figure 6:**
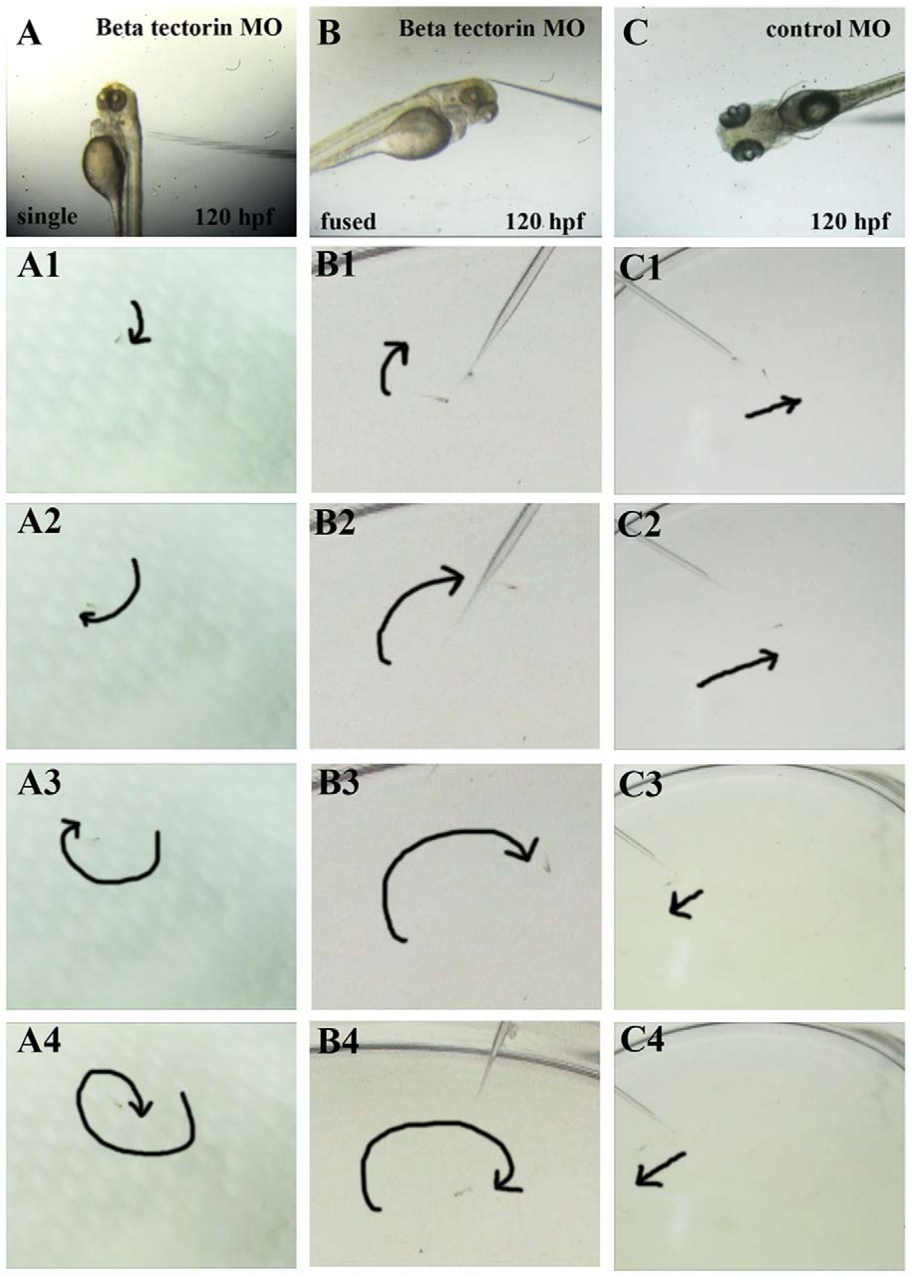
Abnormal swimming behaviors of *β-tectorin* morphants. *β-Tectorin* morphants were examined for their abilities to remain balance and react to a stimulus. Tactile stimulation was created by poking a zebrafish on the head with a glass tube: *β-tectorin* morphants with a single (panel A) and a fused otoliths (panel B), and a control with normal otoliths (panel C). Swimming behaviors of *β-tectorin* morphants at 5 days post-fertilization under stimulation were recorded with a digital video camera. *β-Tectorin* morphants with either single or fused otoliths failed to maintain their balance, tended to remain leaning on one side, remained on the bottom (panel A, B), and tended to swim in a corkscrew (panel A, A1 to A4) or circular manner (panel B, B1 to B4). Control zebrafish maintained their balance, had immediate responses to stimulation, and swam in a straight line (panel C, C1 to C4). The trails of the zebrafish movement were illustrated by dark arrows.

Altered swimming behaviors and a lack of balance are indices of abnormal ear function, for example, swimming in a corkscrew or circular path [21]. Those *β*-*tectorin* morphants with either fused otoliths or a single otolith were further tested for their ability to maintain balance and swim after stimulation. At 5 dpf, about 40% of noninjected embryos (WT) and control MO-injected embryos (N = 70) displayed floating in an upright position and sometimes swam spontaneously in random directions. Another 45% of control MO-injected embryos remained lying down on the bottom of the Petri dish. In order to test whether these 5-dpf zebrafish could respond to vibration, several short vibrations were created by deploying an ultrasonic processor in the water, and all zebrafish swam away immediately (Video S1). The remaining 15% of control MO-injected embryos were further stimulated using a glass tube to touch the head of a zebrafish, and they swam away in a straight manner (Video S3). On the other hand, *β*-*tectorin* morphants with single or fused otoliths showed a failure to maintain balance, and all stayed at the bottom of the Petri dish. About 10% of those *β*-*tectorin* morphants responded to short vibrations created by the ultrasonic processor in the water with short irregular movements (*n* = 70) (Video S2). The remaining *β*-*tectorin* morphants with fused otoliths were stimulated on the head with a glass tube about 5 times, and they either did not respond to the stimuli or swam a very short range in a circular path (Fig. 6, B1 to B4) (Videos S4 and S5). *β*-*Tectorin* morphants with a single otolith had similar behavioral defects as those with fused otoliths described above. Some swam in a corkscrew path which implied profound defects (Fig. 6, A1 to A4) (Videos S6 and S7).

## Discussion

In this study, the zebrafish *β-tectorin* gene and its cDNA were cloned and characterized. The cDNA encodes a protein of 336 amino acids, which displays 49% and 50% identities to human and chick β-tectorins. RT-PCR analyses showed that zebrafish *β-tectorin* mRNA was primarily expressed in the brain with moderate expression in the kidneys. Whole-mount *in situ* hybridization showed that expression of the *β*-*tectorin* transcript was specifically found in the anterior and posterior maculae of the ear. Similar to human β-tectorin, zebrafish β-tectorin contains 4 N-linked glycosylation sites (Fig. 1). Knockdown of zebrafish β-tectorin expression caused the fusion of 2 otoliths or there was only a single otolith, both of which led to severe malfunction of the inner ear.

The predicted amino acid sequence of zebrafish β-tectorin exhibited overall identities of 49%, 50%, 50%, and 50% to β-tectorins from the human, mouse, chick, and *Xenopus* (Fig. 1). However, chick and mouse β-tectorins were homologous with 75% identity at the amino acid level [13], [22]. The higher similarity in identity of chick β-tectorin to the human and mouse compared to zebrafish may be due to differences in habitat, terrestrial and aquatic, respectively. This suggests that the environment may influence the evolution of this molecule. If fish β-tectorins from different species were compared, these fish β-tectorins might display higher identities to each other. Indeed, zebrafish β-tectorin showed higher identities of 74%, 76%, and 74%, with 85% similarity, to β-tectorin from Tetraodon, fugu, and medaka (Fig. S4).

In this study, zebrafish β-tectorin contained a highly conserved ZP domain, which was a sequence of approximately 260 amino acid residues with 8 or 10 cysteine residues and was located at the C-terminus (Fig. 1). Many ZP domain-containing proteins with various functions were found in vertebrates [1]. Some of those proteins constitute the extracellular coat of animal eggs, such as ZP1, ZP2 and ZP3. They are responsible for egg/sperm recognition as well as for blocking polyspermy [23]. Other proteins like α- and β-tectorins are 2 major components of the tectorial membrane, which is an extracellular matrix covering the sensory epithelia of the cochlea of the inner ear [24]. In transgenic mice with a specific mutation in α-tectorin, the structure of the tectorial membrane is disrupted leading to hearing loss [25]. Similarly, mice lacking β-tectorin have sharpened cochlear tuning leading to low-frequency hearing loss [7]. Interestingly, zebrafish β-tectorin is not expressed as a tectorial membrane in the cochlea; instead, it is expressed in anterior and posterior maculae of the zebrafish ear (Fig. 3B), which is similar to the expression of *Starmaker* mRNA [16]. MO knockdown of *β-tectorin* expression affected otolith formation in zebrafish larvae (Fig. 4A). These *β-tectorin* morphants showed a failure to maintain balance and float. Only a few *β*-*tectorin* morphants (10%) were able to respond to a vibration created by an ultrasonic processor in the water, while most of them continued to lie on the bottom of the Petri dish (Video S2). In addition to their responses to short vibrations, *β*-*tectorin* morphants with either fused or single otoliths also showed abnormal swimming patterns after tactile stimulation (Videos S5 and S7). These phenotypes suggest that zebrafish *β-tectorin* has crucial roles in the development and function of the zebrafish inner ear.

Some extracellular matrix proteins are reported to play important roles in the development of the zebrafish inner ear. For instance, MO knockdown of specific genes like *omp-1* and *otolin-1*, which respectively encode otolith matrix protein 1 and a collagen-like protein [19], also showed abnormal otolith formation and impaired swimming behaviors. The omp-1 MO resulted in a reduced otolith size, while otolin-1 MO caused fusion of the 2 otoliths. Therefore, omp-1 was proposed to play important roles in normal otolith growth, while otolin-1 is involved in stabilizing the otolith matrix. In this study, *β-tectorin* morphants also showed similar fused otoliths, but the zebrafish β-tectorin is not a collagen-like protein. Therefore, both otolin-1 and β-tectorin may interact with each other and polymerize into the otolith matrix.

In addition, the expression patterns of *starmaker* and *β-tectorin* mRNAs were similar in the inner ear and in anterior and posterior maculae of the ear as shown in Figs. 3B and 5A. The zebrafish Starmaker protein is a 66-kD protein that is enriched in strongly acidic amino acid residues and 35% proteins, and is also extremely hydrophilic [16]. During zebrafish development, the Starmaker protein is required for otolith biomineralization, and *starmaker* morphants showed starry or chunky otoliths with improper balance in freely swimming zebrafish larvae. Expression of the *starmaker* transcript was slightly reduced in the anterior macula in zebrafish *β-tectorin* morphants with fused otoliths. However, in zebrafish *β-tectorin* morphants with a single otolith, a large portion of the *starmaker* signal was lost in the anterior macula. Interactions among omp-1, β-tectorin, and Starmaker proteins might be the foundation of proper otolith formation.

We also studied the expression of *zpDL1* mRNA in zebrafish *β-tectorin* morphants. *ZpDL1* can be used as a marker to label the 3 sensory cristae of zebrafish embryos at 4 dpf. As shown in Fig. 5C, *zpDL1* signals were lost in lateral cristae of zebrafish *β-tectorin* morphants. These data suggest that zebrafish β-tectorin not only regulates anterior macula formation but is also involved in the morphogenesis of cristae. However, the underlying mechanisms require further investigation.

## Materials and Methods

### Zebrafish care

Zebrafish embryos were raised at 28.5°C, and different developmental stages were determined based on criteria described in the *Zebrafish Book* [26]. All animal procedures were approved by the Animal Use and Care Committee of Academia Sinica (protocol #10-12-114).

### Total RNA isolation and reverse-transcription polymerase chain reaction (RT-PCR) analysis of zebrafish *β*-*tectorin* mRNA

Total RNA was isolated from different developmental stages and various tissues of adult zebrafish, using the RNAzol reagent (Tel-Test, Friendswood, TX, USA) according to the instructions of the manufacturer. After treatment with RQ1 RNase-Free DNaseI (Promega, Madison, WI, USA), 50∼100 µg of total RNA was subjected to the first-strand cDNA synthesis. PCR amplifications were performed with the following zebrafish *β*-*tectorin* RT-PCR primers (*β*-*tectorin*-RT-F, 5′- GCT GCT GAA GAC CTA CAC AGG AAC-3′ and *β*-*tectorin*-RT-R, 5′-TGG ATG TAT GCA TGC ATG CGT GTC-3′). Zebrafish β-actin primers (zACT-F, 5′-GTG CTA GAC TCT GGT GAT GGT GTG-3′ and zACT-R, 5′-GGT GAT GAC CTG ACC GTC AGG AAG-3′) were used for the internal control to amplify a DNA fragment using cDNA as a template. Primers for examining the efficiency of the splice MOs are as follow (P1, 5′- GCT GCT GAA GAC CTA CAC AGG AAC- 3′; P2, 5′ -GGC TAA ACA CGG CGT TGT TGA CCA- 3′.)

### Cloning of the full-length cDNA encoding zebrafish β-tectorin

To identify zebrafish complementary (c)DNA related to the human *β-tectorin* gene, we used the coding region of human *β-tectorin* (accession no. XM_521604) to search GenBank for related expression sequence tags (ESTs) using the tBLAST program and found some zebrafish EST clones (CN315850 and EG585664) related to human *β-tectorin*. Using 5′- and 3′-RACE to obtain the 5′- and 3′-untranslated regions (UTRs), we assembled all sequences to obtain 1542-bp cDNA with an open reading frame (ORF) of 1011 bp encoding a protein of 336 amino acid residues. The complete sequence was deposited in GenBank with the accession number of FJ374270.

The full-length cDNA encoding zebrafish *β*-*tectorin* was isolated by PCR amplification using gene-specific primers with linkers (*β*-*tectorin*-BamH1-F, 5′-CC GGA TCC ATG GCA GCT GTT GGC CTT-3′, and *β*-*tectorin*-EcoR1-R, 5′-GG GAA TTC AAA AGT AAA GTA TCC TAA-3′) according to the sequence submitted with GenBank accession no. FJ374270. Full-length zebrafish *β*-*tectorin* was subcloned into the BamH1 and EcoR1 sites of pcDNA3.1-myc to generate pcDNA3.1-*β*-*tectorin*- myc. Full length *β*-*tectorin* was then further subcloned to T7TS plasmid using BamH1 and Xba1 sites.

### Rescue of defects in *β*-*Tectorin* morphant by injecting *β*-*Tectorin* RNA

Full length *β*-*Tectorin* was cloned into T7TS plasmid and synthesized *in vitro*. T7TS-*β*-*Tectorin* was linearized to synthesize capping mRNA by using mMESSAGE mMACHINE T7 Kit (Ambion, Foster City, CA, USA). ∼100 pg of *β*-*Tectorin* RNA was injected into embryos at the one- to two-cell stage.

### Morpholino oligonucleotide (MO) injection

Antisense MOs were obtained from Gene Tools (Philomath, OR, USA), and the sequence of zebrafish *β*-*tectorin* MO was as follows: 5′-GTG GCA GAA TCC AGA AGA AAT GTT G-3′. The sequence of the two splice MOs used were as follow: splice MO 1 : 5′- AAC CCA TCA AAC ATC TTA CCT CAG A-3′ and splice MO 2 : 5′-CCT CCT ACA TAC TGA AAA GAA GGT A-3′. The morpholinos were resolved to 24 µg/µl injection stock, and stored in a −20°C refrigerator. The diluted morpholino was injected into wild-type (WT) zebrafish embryos at the 1∼2-cell stage using a microinjection system consisting of an SZX9 stereomicroscope (Olympus, Tokoyo, Japan) and an IM300 Microinjector (Narishige, Tokoyo, Japan). The sequence of p53 MO was as follow (p53 MO: 5′-AAA ATG TCT GTA CTA TCT CCA TCC G-3′) [27].

To confirm the specificities of the *β*-*tectorin* morpholino, several pCMV-GFP reporter plasmids were created. The morpholino targeted a 25-bp sequence of the PCR by the following primer pairs for the perfect match, bTec-GFP (bTec-GFP-F, 5′- GAT CCC AAC ATT TCT TCT GGA TTC TGC CAC G-3′ and bTec-GFP-R, 5′-AAT TCG TGG CAG AAT CCA GAA GAA ATG TTG G-3′). For a 5-base exchanged mismatch, MM-b-Tec-GFP was used (MM-b-Tec-GFP-F, 5′-GAT CCG AAG ATT ACT TCT GCA TTC TGG CAC G-3′ and MM-b-Tec-GFP-R,5′-AAT TCG TGC CAG AAT GCA GAA GTA ATC TTC G-3′). The 5′ region of the zebrafish *β*-*tectorin* mRNA was fused to the N-terminal of the GFP protein. Either construct bTec-GFP or MM-b-Tec-GFP was co-injected with zebrafish *β*-*tectorin* morpholino, and the fluorescence was analyzed by a fluorescent microscope at 48 hpf.

### Whole-mount *in situ* hybridization

Digoxigenin-labeled RNA probes (Roche, Penzberg, Germany) were generated by *in vitro* transcription using the linearized pGEM-T-easy plasmids (Promega, Madison, WI, USA) carrying the 3′-UTR of the following zebrafish genes. Whole-mount *in situ* hybridization was performed following a previously described protocol [28]. Specific primers for *stm* (stm-F, 5′- GAA TCA ACT GAG ACA GTC AAG ATA ACC -3′ and stm-R, 5′- TGA GAG TGG AGA GCG GGA ATT ATC TGC - 3′), *zpDL1* ( zpDL1-F, 5′-GCG GGA CAT CAG TGT GTA TTG TGG AGT TCA -3′ and zpDL1-R, 5′- GCA AGC TGT GTG TTG TTG ACC AGG TAT TCC -3′), and omp-1 (zomp-1-F, 5′- CAC ACT ACA GTC TTT GAC AAC ATG - 3′ and zomp-1-R, 5′- CAT CAG ATC AAC ACA AAC CTT CAC - 3′) were used to amplify the 3′-UTR of each gene. Primers used in the RT-PCR of zebrafish *β*-*tectorin* were also used to make the zebrafish *β*-*tectorin* probe.

### FM1-43 labeling of hair cells

Labeling the hair cells in the inner ear with 40 µM of FM1-43 (N-(3-triethylammoniumpropyl)-4(4-(dibutylamino)styryl) pyridinium dibromide), (Invitrogen, Carlsbad, CA, USA) dissolved in the extracellular solution. An injection tube was used to inject FM1-43 into the otic vesicle following the protocols described previously [29]. The formula of extracellular solution is described as follow, 134 mM NaCl, 2.9 mM KCl, 1.2 mM MgCl_2_, 2.1 mM CaCl_2_, 10 mM HEPES, and 10 mM glucose, and was adjusted to pH 7.8.

### Video recording of the swimming behavior of zebrafish *β*-*tectorin* morphants

Zebrafish *β*-*tectorin* morphants were recorded with a digital video camera (Sony DCR-PC120 digital camera, Tokyo, Japan) at 5 dpf to examine their reactions to short vibrations and tactile stimulation. Short vibrations were created by a Hielscher Up50H ultrasonic processor (Hielscher, Teltow, Germany), at 30 kHz and 50 W with an amplitude of 30% and 0.5 s per cycle. Tactile stimulation was created using a glass tube to touch the head of a zebrafish, and instantaneous responses were recorded with a digital camera. Zebrafish *β*-*tectorin* morphants and control MO-injected zebrafish were touched on the head at least 5 times for each test. The swimming behavior of the zebrafish was observed and defined by whether the fish swam in a straight or circular manner.

## Supporting Information

Figure S1 **Control experiments for morpholino specificity.** To determine the specificities of the morpholinos used, pCMV-GFP reporter plasmids containing a perfect (bTec-GFP) or mismatched (MM-b-Tec-GFP) MO target sequence were employed. Both bTec-GFP (A) and MM-b-Tec-GFP were co-injected with the *β-tectorin* MO. All images were taken from zebrafish embryos at 48 h post-fertilization.
(TIF)

Figure S2 **The splice MO targeting and RT-PCR analysis of** ***β*** **-tectorin mRNAs of embryos injected with splice MOs.** (A) The exon-intron genomic structure from exons 1–4 was shown. Splice MO 1 and MO 2 target the donor and acceptor sites, respectively. (B) Total RNAs were extracted from control MO (C) and splice MO1/MO2-injected (MO) embryos at 72 hpf, then RT-PCR was performed. Primers (P1/P2) flanking the region resulted in a single 500 bp band in the case of control embryos. On the other hand, in the case of morphants, the level of this band was strongly reduced and a second 1800 bp band was visible. The second band resulted from the use of an alternative splice donor. β-Actin bands were used to normalize the amount of cDNA prepared from both embyos.
(TIF)

Figure S3 **The morphology of** ***β*** **-tectorin morphants.** The ATG MO injected zebrafish *embryos* with fused (a, b), and single otoliths (c, d) appeared to be normal without obvious defects. All photographs were taken at 72 hpf.
(TIF)

Figure S4 **Zebrafish β-tectorin amino acid sequence alignment with other fish species.** The deduced amino acid sequences of zebrafish β-tectorin were aligned with those from Tetraodon, fugu, and medaka. Identical residues in 3 or 4 proteins are highlighted. The accession numbers of each *β*-tectorin from different fish species are listed below: Tetraodon (GenBank, accession no: CAG06543), fugu (ensembl no: ENSTRUP00000021095), and medaka (ensembl no: ENSORLP00000014650).
(TIF)

Video S1 **Control MO-injected zebrafish responded to vibrations made with an ultrasonic processor.** Control MO-injected zebrafish at 5 days post-fertilization (dpf) with normal otoliths were placed in a Petri dish, and a transient vibration was generated with a Hielscher Up50H ultrasonic processor, at 30 kHz and 50 W with an amplitude of 30% and 0.5 s per cycle.
(WMV)

Video S2 **The** ***β-tectorin*** **morphant responded to the vibration made with an ultrasonic processor.** The *β-tectorin* morphant at 5 days post-fertilization with either single or fused otoliths were placed in a Petri dish, and a transient vibration was generated with a Hielscher Up50H ultrasonic processor.
(WMV)

Video S3 **Response of control MO-injected zebrafish to touch with a glass tube.** Control MO-injected zebrafish at 5 days post-fertilization with normal otoliths was touched on the head with a glass tube at least 5 times, and its swimming behavior was observed. The bright-field videomicrograph was taken with an Olympus IX70-FLA inverted fluorescence microscope equipped with a Sony DCR-PC120 digital video camera.
(WMV)

Video S4 **Response of a** ***β-tectorin*** **morphant with fused otoliths to touch with a glass tube.** The *β-tectorin* morphant at 5 days post-fertilization with fused otoliths was touched on the head with a glass tube at least 5 times. The bright-field videomicrograph was taken with an Olympus IX70-FLA inverted fluorescence microscope equipped with a Sony DCR-PC120 digital video camera.
(WMV)

Video S5 **Swimming behavior of a** ***β-tectorin*** **morphant with fused otoliths.** The *β-tectorin* morphant at 5 days post-fertilization (dpf) with fused otoliths was touched on the head with a glass tube at least 5 times, and its swimming behavior was observed. The video was taken with a Sony DCR-PC120 digital video camera.
(WMV)

Video S6 **Response of a** ***β-tectorin*** **morphant with a single otolith to touch with a glass tube.** The *β-tectorin* morphant at 5 days post-fertilization with a single otolith was touched on the head with a glass tube at least 5 times. The bright-field videomicrograph was taken with an Olympus IX70-FLA inverted fluorescence microscope equipped with a Sony DCR-PC120 digital video camera.
(WMV)

Video S7 **Swimming behavior of a** ***β-tectorin*** **morphant with a single otolith.** The *β-tectorin* morphant at 5 days post-fertilization with a single otolith was touched on the head with a glass tube at least 5 times, and its swimming behavior was observed. The video was taken with a Sony DCR-PC120 digital video camera.
(WMV)
