# Correction: Zona Pellucida Domain-Containing Protein β-Tectorin is Crucial for Zebrafish Proper Inner Ear Development

**DOI:** 10.1371/annotation/7f7eb48c-7429-4119-89de-9c9bc3d85f04

**Published:** 2012-02-28

**Authors:** Chung-Hsiang Yang, Chia-Hsiung Cheng, Gen-Der Chen, Wei-Hao Liao, Yi-Chung Chen, Kai-Yun Huang, Pung-Pung Hwang, Sheng-Ping L. Hwang, Chang-Jen Huang

There was an error in the accession number in the results section titled "Development of the inner ear was affected in the β-tectorin MO morphants as shown by whole-mount in situ hybridization." The correct accession number is XM_001921951.2.

