# Supplementary figures and images for: Zona Pellucida Domain-Containing Protein β-Tectorin is Crucial for Zebrafish Proper Inner Ear Development
Source: PLoS One. 2011 Aug 2;6(8):e23078. doi: 10.1371/journal.pone.0023078 (PMC3149068; doi:10.1371/journal.pone.0023078)

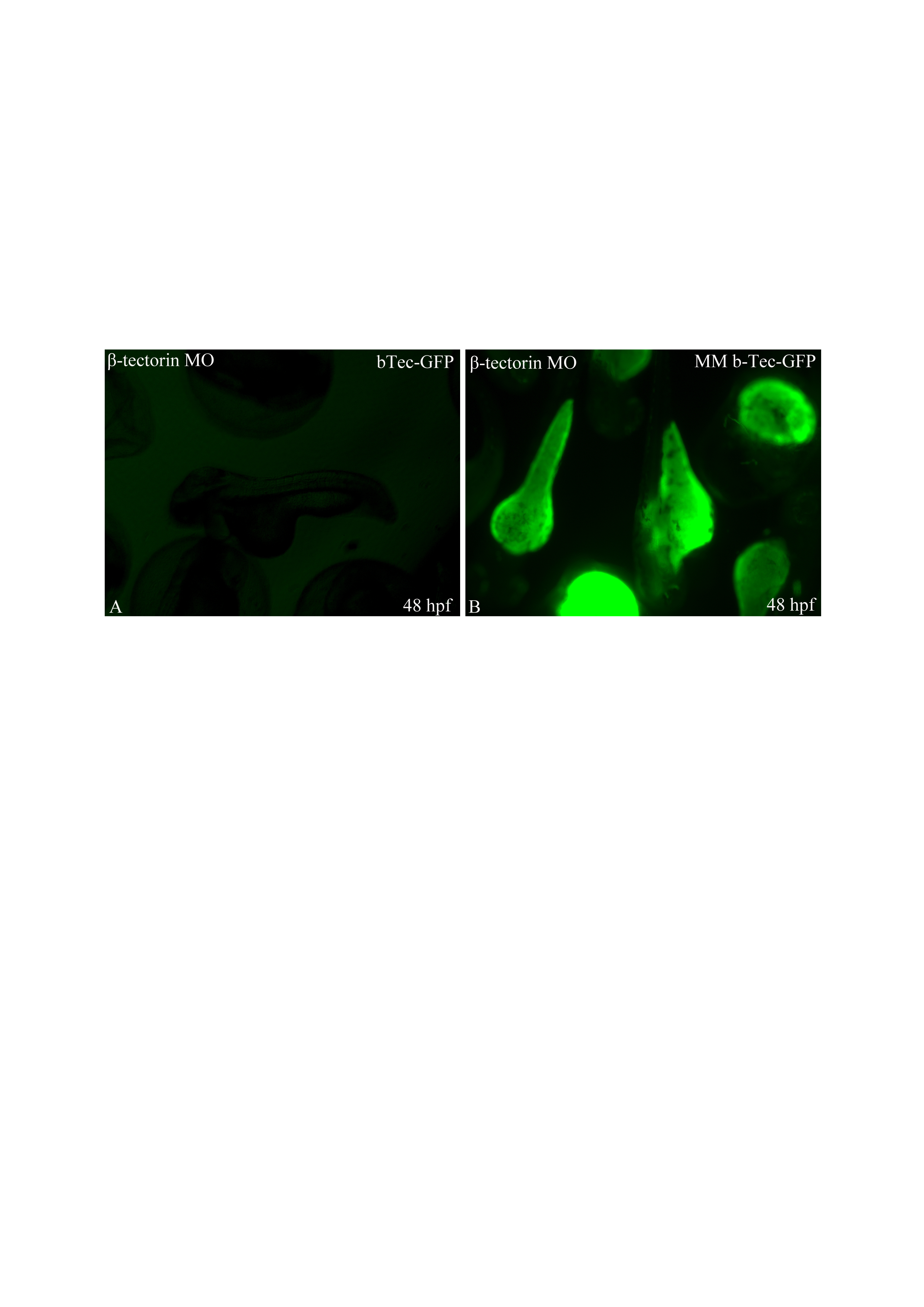

Supplement: Figure S1 — Control experiments for morpholino specificity. To determine the specificities of the morpholinos used, pCMV-GFP reporter plasmids containing a perfect (bTec-GFP) or mismatched (MM-b-Tec-GFP) MO target sequence were employed. Both bTec-GFP (A) and MM-b-Tec-GFP were co-injected with the β-tectorin MO. All images were taken from zebrafish embryos at 48 h post-fertilization. (TIF) [file pone.0023078.s001.tif]

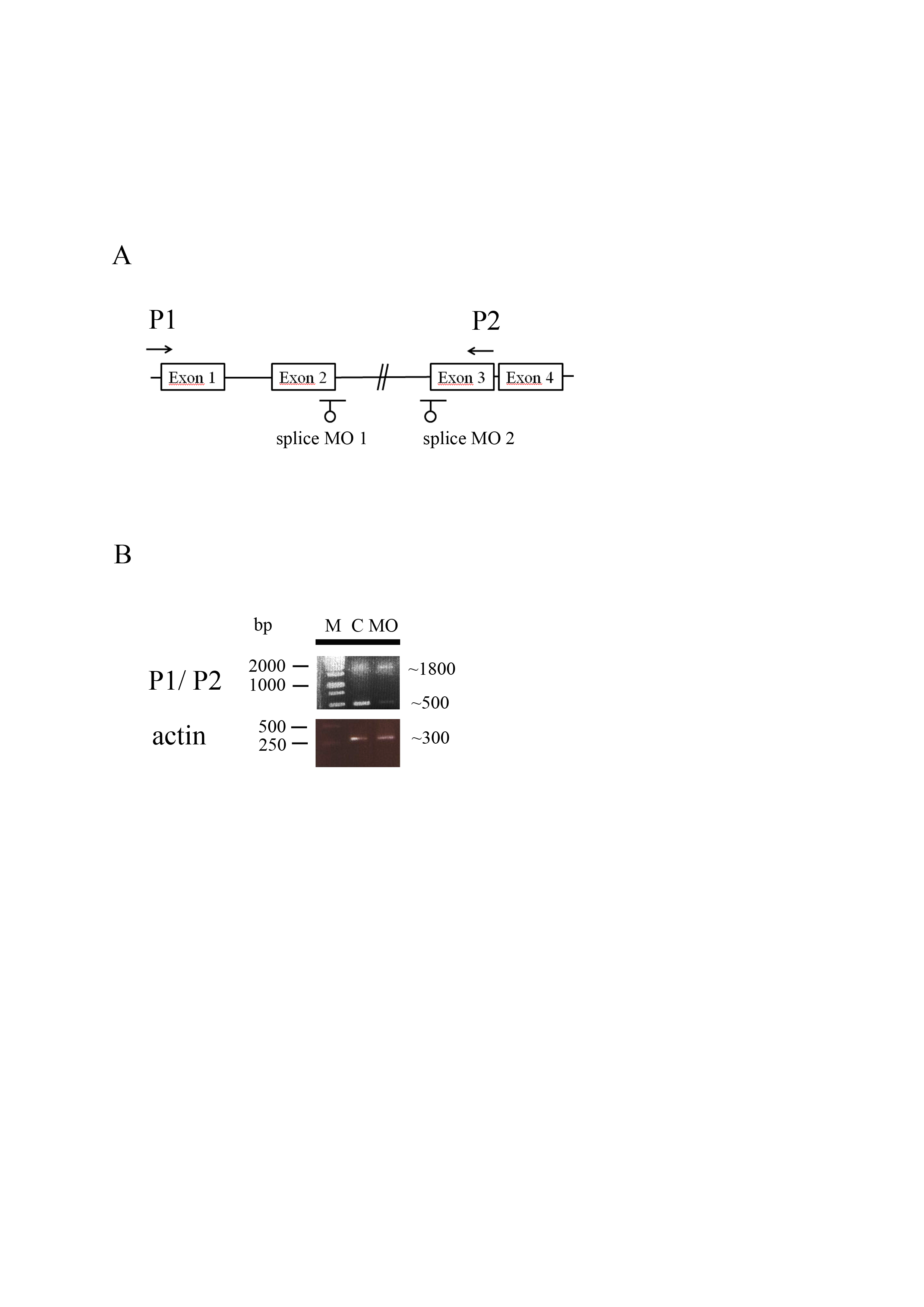

Supplement: Figure S2 — The splice MO targeting and RT-PCR analysis of β -tectorin mRNAs of embryos injected with splice MOs. (A) The exon-intron genomic structure from exons 1–4 was shown. Splice MO 1 and MO 2 target the donor and acceptor sites, respectively. (B) Total RNAs were extracted from control MO (C) and splice MO1/MO2-injected (MO) embryos at 72 hpf, then RT-PCR was performed. Primers (P1/P2) flanking the region resulted in a single 500 bp band in the case of control embryos. On the other hand, in the case of morphants, the level of this band was strongly reduced and a second 1800 bp band was visible. The second band resulted from the use of an alternative splice donor. β-Actin bands were used to normalize the amount of cDNA prepared from both embyos. (TIF) [file pone.0023078.s002.tif]

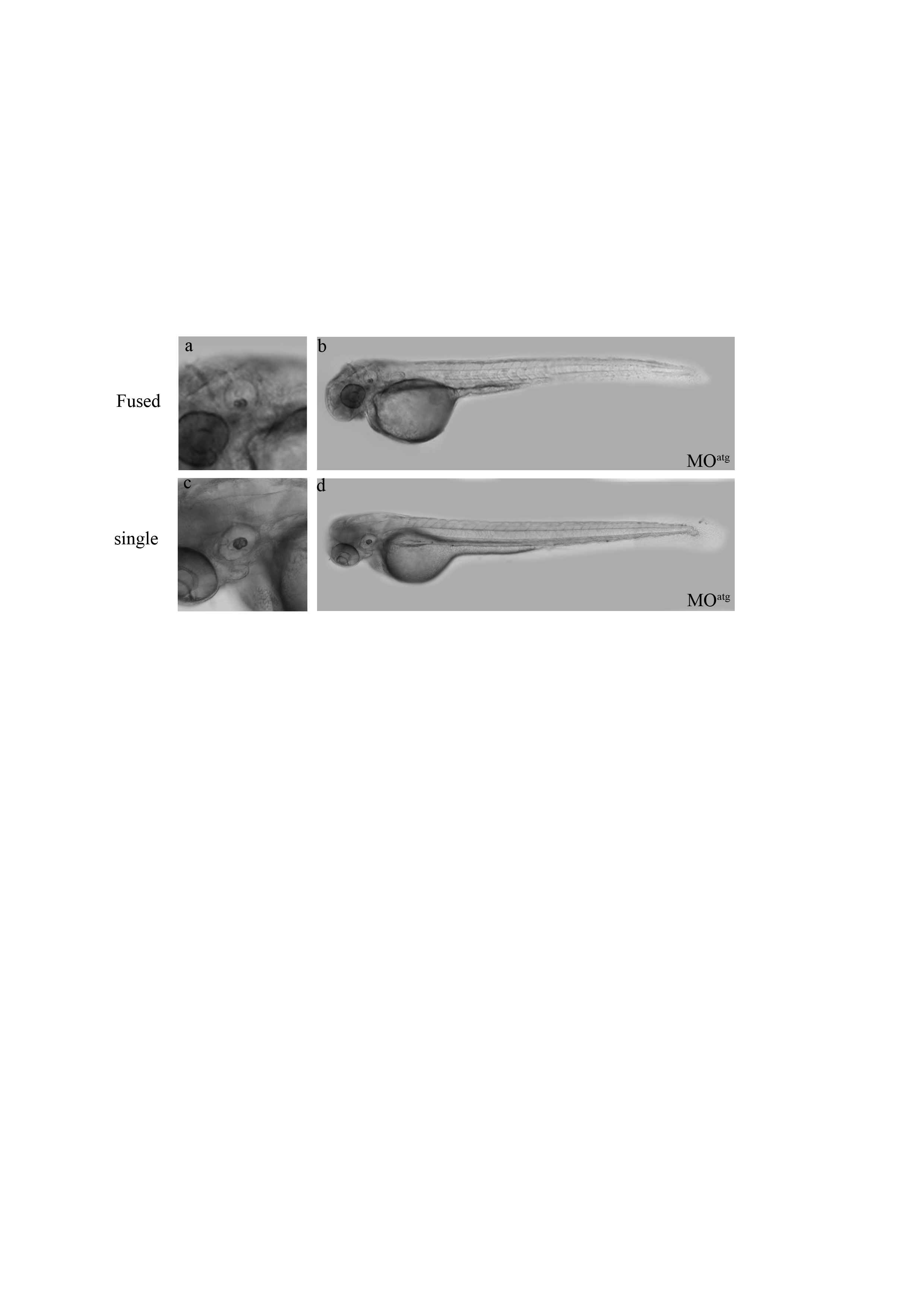

Supplement: Figure S3 — The morphology of β -tectorin morphants. The ATG MO injected zebrafish embryos with fused (a, b), and single otoliths (c, d) appeared to be normal without obvious defects. All photographs were taken at 72 hpf. (TIF) [file pone.0023078.s003.tif]

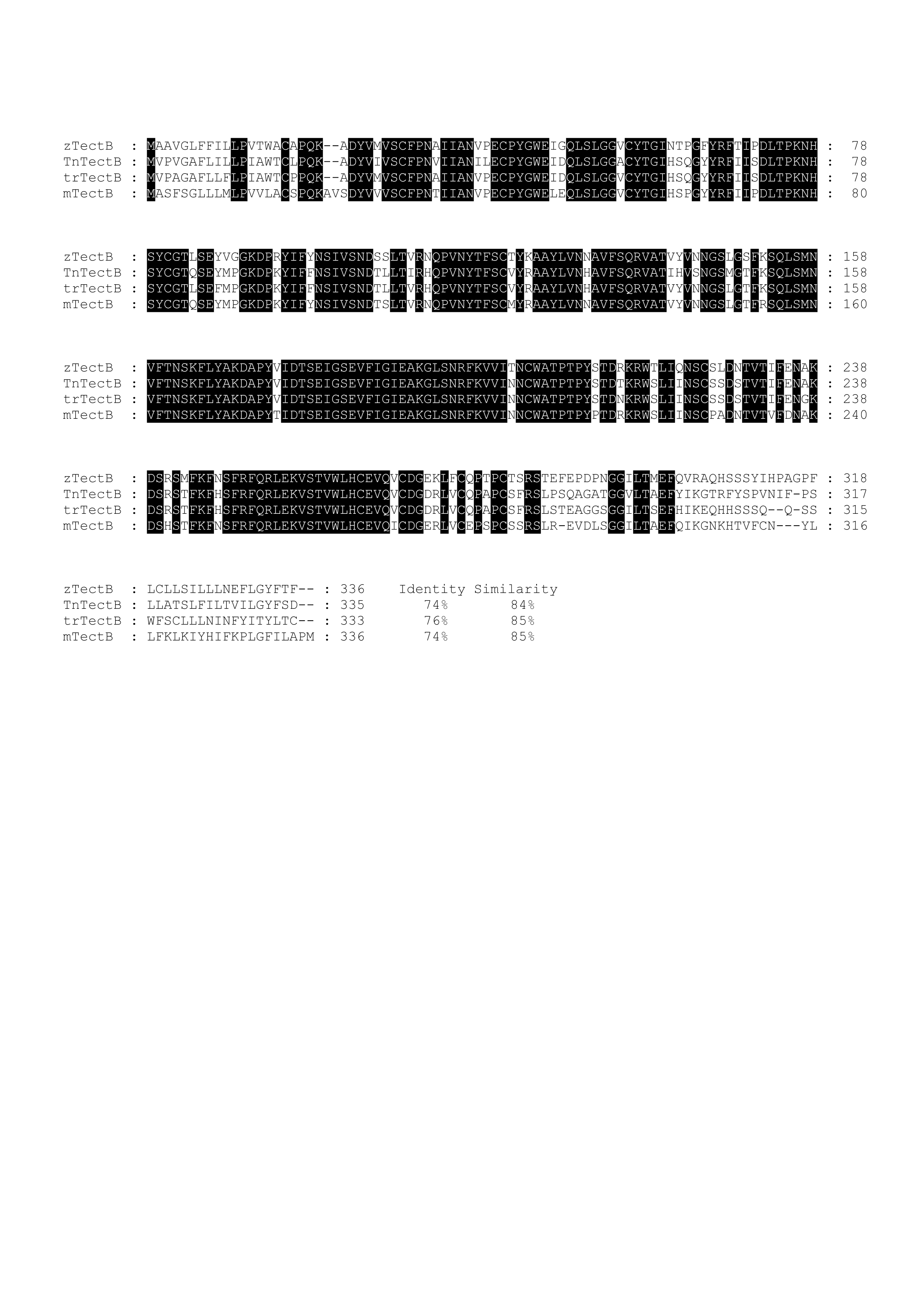

Supplement: Figure S4 — Zebrafish β-tectorin amino acid sequence alignment with other fish species. The deduced amino acid sequences of zebrafish β-tectorin were aligned with those from Tetraodon, fugu, and medaka. Identical residues in 3 or 4 proteins are highlighted. The accession numbers of each β-tectorin from different fish species are listed below: Tetraodon (GenBank, accession no: CAG06543), fugu (ensembl no: ENSTRUP00000021095), and medaka (ensembl no: ENSORLP00000014650). (TIF) [file pone.0023078.s004.tif]
